# Micromechanical Modeling of the Elasto-Viscoplastic Behavior and Incompatibility Stresses of β-Ti Alloys

**DOI:** 10.3390/ma11071227

**Published:** 2018-07-17

**Authors:** Safaa Lhadi, Maria-Rita Chini, Thiebaud Richeton, Nathalie Gey, Lionel Germain, Stéphane Berbenni

**Affiliations:** 1 Laboratoire d’Étude des Microstructures et de Mécanique des Matériaux, Université de Lorraine, CNRS, Arts et Métiers Paris Tech, LEM3, F-57000 Metz, France; safaa.lhadi-khalouche@univ-lorraine.fr (S.L.); rita.chini@univ-lorraine.fr (M.-R.C.); thiebaud.richeton@univ-lorraine.fr (T.R.); nathalie.gey@univ-lorraine.fr (N.G.); lionel.germain@univ-lorraine.fr (L.G.); 2 Laboratory of Excellence on Design of Alloy Metals for low-mAss Structures (DAMAS), Université de Lorraine, F-57000 Metz, France

**Keywords:** polycrystalline β-Ti, elastic anisotropy, elastic/plastic incompatibilities, elasto-viscoplastic self-consistent scheme (EVPSC), slip activity

## Abstract

Near β titanium alloys can now compete with quasi-α or α/β titanium alloys for airframe forging applications. The body-centered cubic β-phase can represent up to 40% of the volume. However, the way that its elastic anisotropy impacts the mechanical behavior remains an open question. In the present work, an advanced elasto-viscoplastic self-consistent model is used to investigate the tensile behavior at different applied strain rates of a fully β-phase Ti alloy taken as a model material. The model considers crystalline anisotropic elasticity and plasticity. It is first shown that two sets of elastic constants taken from the literature can be used to well reproduce the experimental elasto-viscoplastic transition, but lead to scattered mechanical behaviors at the grain scale. Incompatibility stresses and strains are found to increase in magnitude with the elastic anisotropy factor. The highest local stresses are obtained toward the end of the elastic regime for grains oriented with their <111> direction parallel to the tensile axis. Finally, as a major result, it is shown that the elastic anisotropy of the β-phase can affect the distribution of slip activities. In contrast with the isotropic elastic case, it is predicted that {112} <111> slip systems become predominant at the onset of plastic deformation when elastic anisotropy is considered in the micromechanical model.

## 1. Introduction

Near-β titanium alloys are constituted of hexagonal compact (HCP) α-phase and body-centered cubic (BCC) β-phase, with the latter representing up to 40% of the volume. These alloys can achieve high specific strength thanks to transformation processes, including β and α/β forging before aging. They can now compete with quasi-α or α/β titanium alloys for airframe forging applications such as landing gears, turbine engines, and rotor systems. The microstructure of near-β titanium alloys is complex and multiscale. The α-phase contains primary α nodules and secondary α platelets that are embedded in β grains. It has been recently shown that the α/β forging process does not completely break the prior millimeter-sized β forged grains, but rather fragments them into equiaxed subgrains with close orientations and diameters about 5 µm to 10 µm [1].

The impact of the β-phase on the mechanical properties of near-β titanium alloys has not been completely understood yet. The β-phase (BCC) is elastically anisotropic [2] so that the elastic behavior of β-phase single crystal is strongly dependent on the loading direction. Owing to its cubic symmetry, the β-single crystal elastic stiffness tensor contains three independent elastic constants C_11_, C_12_, and C_44_. The degree of elastic anisotropy of such a cubic phase can be evaluated by the so-called Zener factor [3], which is defined as:

(1) $$A=\frac{2C_{44}}{C_{11}-C_{12}}$$

The published single-crystal elastic constants (SEC) for the β-phase span very different values, and the reported Zener anisotropy factor A ranges from 1.4 to 8.3 [4,5,6,7,8,9,10,11,12,13]. These discrepancies may be due to the specific chemical compositions and thermomechanical treatments of the studied Ti alloys, but also to the difficulty to measure SEC in multiphase polycrystalline specimens.

It is well known that microstructural features such as grain size, crystallographic texture, and grain morphology strongly influence the mechanical properties of polycrystalline materials [14,15,16]. In addition, the elastic anisotropy may also influence the elastic-viscoplastic transition (microplastic regime, incipient plasticity…) and the development on internal stresses, which might initiate cracks [17]. However, only a few studies have focused on the effect of the β-phase elastic anisotropy in near-β Ti alloys [4,5,18,19,20]. Therefore, the purpose of the present paper is to perform a comprehensive study of the role of the β-phase elastic anisotropy on the local and overall mechanical behavior of a fully β Ti alloy taken as a model material. Tensile tests performed at different strain rates on Ti-17 alloys with 100% equiaxed β microstructures constitute the experimental basis of the study [4,21,22]. On the other hand, the theoretical investigation is carried out through the use of an advanced elasto-viscoplastic self-consistent model (EVPSC) that considers an affine linearization of the viscoplastic flow rule and one-site self-consistent approximation thanks to the translated field method [23]. It is thought that such a micro–macro scale transition model is well suited to the modeling of disordered materials such as fully β-phase Ti polycrystalline aggregates. Indeed, the EVPSC model can account for crystal elasticity and plasticity, crystallographic texture effects, and strain-rate effects, unlike the elastoplastic self-consistent (EPSC) scheme [5]. Moreover, it is able to consider an extensive number of grains described by their crystallographic orientations, volume fractions, and morphologies. As outputs, it provides the local (at the scale of the grains) and overall effective (at the scale of the polycrystalline aggregate) mechanical behaviors. This study aims to better understand the effects of the elastic anisotropy on the strain-hardening behavior, the local mechanical fields, and the distribution of slip activities.

The paper is organized as follows. Section 2 is devoted to a brief description of the micromechanical model. The governing field equations of the elasto-viscoplastic problem are reviewed, and the single crystal constitutive laws are described. Model parameters and results are reported and discussed in Section 3. Finally, concluding remarks are summarized in Section 4.

## 2. Micromechanical Model

In this paper, the micro–macro scale transition from single to polycrystal is assessed through the elasto-viscoplastic self-consistent (EVPSC) model based on the translated field method and on an “affine” extension of the viscoplastic strain rate first introduced in [23] for thermo-elasto-viscoplastic heterogeneous materials. Here, thermal effects (i.e., thermal strains) are disregarded. The used EVPSC model has been described in details elsewhere [20,23,24], and therefore, only a brief description is given here.

### 2.1. Fields Equations

In homogenization theory, the macroscopic stress rate and strain rate tensors $(\dot{\Sigma }$ and $\dot{E})$ of a representative volume element (RVE) with volume V are obtained by volume averaging the local (grain) stress rate and strain rate tensors $(\dot{\sigma }$ and $\dot{\varepsilon })$ as follows:

(2) $$\dot{\Sigma }=\langle \dot{\sigma }\rangle ,\;\dot{E}=\langle \dot{\varepsilon }\rangle$$

Within the infinitesimal strain framework, the total local strain rate relative to an elasto-viscoplastic behavior (of Maxwell type) is decomposed into:

(3) $$\dot{\varepsilon }={\dot{\varepsilon }}^{e}+{\dot{\varepsilon }}^{vp}$$

with ${\dot{\varepsilon }}^{e}=s:\dot{\sigma }$ from the generalized Hooke’s law where s $\left(=c^{-1}\right)$ is the local elastic compliance tensor, and ${\dot{\varepsilon }}^{vp}$ is given by a nonlinear viscoplastic constitutive flow rule that depends on the Cauchy stress tensor σ. Static equilibrium in the absence of body force and kinematic compatibility complete the field equations of the problem [23].

### 2.2. Single Crystal’s Constitutive Behavior

The behavior of the β single crystal is supposed to be elastic-viscoplastic, where plastic strain and rotation result from crystallographic slips on specific systems “s”.

The constitutive viscoplastic model with “pseudo-multiplier” developed by Méric et al. (1991) [25] and Méric and Cailletaud [26] is used to describe the slip rate ${\dot{\gamma }}^{s}$ for each slip system s:

(4) $${\dot{\gamma }}^{s}={\langle \frac{\left|\tau^{s}-x^{s}\right|-r^{s}}{Κ}\rangle }^{n}\;\mathrm{sign}\left(\tau^{s}-x^{s}\right)$$

where 〈x〉=max(x,0) and $\tau^{s}$ is the resolved shear stress, $\tau^{s}=R^{s}:\sigma$ with $R^{s}$ is the symmetric Schmid orientation tensor, and $x^{s}$ and $r^{s}$ are associated with kinematic and isotropic hardening on s, respectively:

(5) $$x^{s}=c^{s}\alpha^{s}\;\mathrm{with}\;{\dot{\alpha }}^{s}={\dot{\gamma }}^{s}-d^{s}\alpha^{s}\left|{\dot{\gamma }}^{s}\right|$$

and:

(6) $$r^{s}=r_{0}^{s}+\sum_{r=1}^{N}H_{\mathrm{sr}}b^{r}Q^{r}q^{r}\;\mathrm{with}\;{\dot{q}}^{r}=\left(1-b^{r}q^{r}\right)\left|{\dot{\gamma }}^{r}\right|$$

In the above equations, Κ and n are two material coefficients characterizing the viscous effect, c, b, d and q are hardening parameters, and H is an N×N matrix describing the interactions between the different slip systems, where N is the total number of the slip systems (here N = 48, see Section 3.1) of the β single crystal. Therefore, at the grain level, the viscoplastic strain rate is given by:

(7) $${\dot{\varepsilon }}^{vp}=\sum_{s=1}^{N}R^{s}{\langle \frac{\left|\tau^{s}-x^{s}\right|-r^{s}}{Κ}\rangle }^{n}\;\mathrm{sign}\left(\tau^{s}-x^{s}\right)$$

### 2.3. Self-Consistent Approximation Based on Affine Translated Field Method

Here, the one-site self-consistent approximation is formulated using the translated field method [27,28,29], which is an internal variable approach. The details of this method with an affine linearization of the viscoplastic flow rule can be found in [20]. Compared to hereditary approaches [30], the numerical implementation is much easier, as no use of Laplace–Carson transform is needed. As a result, the explicit expressions of the tensors of stress rates $\dot{\sigma }$ and strain rates $\dot{\varepsilon }$ at the level of each grain with a given crystallographic orientation can be obtained as a function of applied stress rates and/or strain rates $(\dot{\Sigma }$ and $\dot{E})$ (mixed boundary conditions) and stress history σ in the grain depending on coupled elastic and viscoplastic intergranular accommodations. For details, the reader can find the stress and strain rate concentration equations of the self-consistent model in [20,23,24].

## 3. Results and Discussions

**Figure 1:**
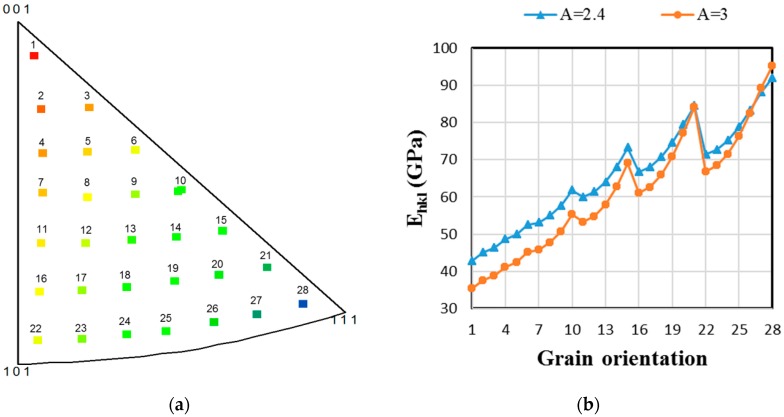
(**a**) Inverse pole figure representing the 2016 grains with random texture decomposed into 28 fiber components with respect to the tensile axis; (**b**) Directional Young’s moduli of single crystals with orientations 1 to 28 parallel to the tensile axis obtained from both considered β-phase single-crystal elastic constants (SEC) (A = 2.4 (triangles) and A = 3 (circles)).

For all of the simulation results shown in this paper, the considered polycrystalline material has a fully β-phase microstructure. Furthermore, the representative volume element (RVE) always contains 2016 equiaxed grains with the same volume fraction and random crystallographic texture. The texture is first decomposed into 28 fiber components parallel to the loading direction. All of the grain orientations are deduced by a rotation of 5° around the tensile axis (see the inverse pole figure in Figure 1a). Besides, in the whole paper, the tensile loading direction is parallel to the $X_{3}$-axis.

### 3.1. Model Parameters

Two sets of β SEC are considered in this study: The ones considered by Martin (2012) [4], which gives a Zener elastic anisotropy factor of A = 2.4, and the ones of Petry et al. (1991) [6], which gives A = 3. As reported in [20], both SEC give rise to effective Young’s modulus close to the measured one for a fully β microstructure Ti-5553 and Ti-17 alloys, which are about 68 GPa and 60 GPa, respectively [21,31]. Table 1 provides the values of the SEC and the corresponding effective Young’s modulus computed for a fully β microstructure assuming equiaxed grains and random texture. Unlike the computed effective Young’s modulus for the polycrystal with isotropic crystallographic orientation distribution, the single crystalline Young’s moduli for the 28 individual orientations are generally different for each SEC (see Figure 1b). As expected, the gap between the directional Young’s moduli of single crystals along the directions <100> (i.e., grain orientation 1) and <111> (grain orientation 28) increases with the anisotropy factor A.

**Figure 2:**
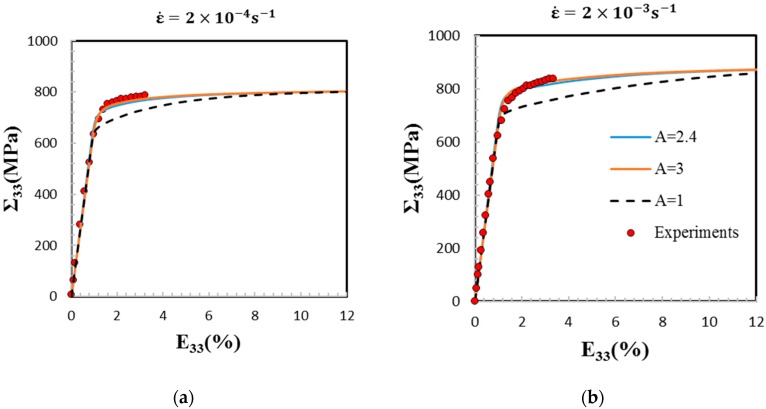
Tensile macroscopic stress ($\Sigma_{33}$) vs. macroscopic strain ($E_{33}$) estimated by the Affine elasto-viscoplastic self-consistent model (EVPSC) model at two strain rates: $2\times 10^{-4}\;s^{-1}\;\left(a\right)\;\mathrm{and}\;2\times 10^{-3}\;s^{-1}\;\left(b\right)$ using three anisotropy factors (A). The numerical results are compared to the experimental data provided in [4,21] (red circles). For the calculations, an equiaxed β microstructure with random texture is considered.

Table 2 provides the viscoplastic model parameters that are related to the β single crystal. The Méric-Cailletaud’s flow rule parameters [26] were identified by [4] on Ti-5553 and Ti-17 both 100% β equiaxed grains with random texture. Here, the experimental yield strengths and the elastic-viscoplastic transition on the tensile stress–strain curves at both applied strain rates (see Figure 2) were used to fit the initial critical resolved shear stresses (CRSS) for the three slip system families of the BCC β single crystal denoted r_0_. The values of r_0_ are reported in Table 2. Furthermore, a quasi-linear hardening rate is observed between 2–4% strain (see Figure 2). Therefore, here, a linear kinematic hardening is sufficient (c ≠ 0, d = 0) for the monotonic tensile test simulations, and the isotropic hardening parameters were disregarded (Q = d = 0). Both material parameters n and K (see Table 2) were identified to reproduce the low strain rate sensitive behavior between 1–4% strains using the experimental points at both strain rates. The elastic anisotropy factor of A = 3 was used to identify the viscoplastic parameters. For further justifications on the choice of these viscoplastic parameters in the constitutive equations for the present EVPSC scheme, the reader can refer to the recent work by Lhadi et al. [20].

### 3.2. Macroscopic Stress–Strain Responses and Strain-Hardening Behavior

Simulation results show that both sets of SEC (Table 1) predict very well the tensile stress–strain curves obtained on 100% β Ti-17 at two strain rates ($2\times 10^{-4}\;s^{-1}$ and $2\times 10^{-3}\;s^{-1}$) [21]. In all of the figures, we refer to the SEC by their respective anisotropy factor. Figure 2 compares the experimental data and the predictions of the present model. As shown in the figure, the elasto-viscoplastic transition is well captured with both A = 2.4 and 3.

The stress–strain curves obtained for the elastic isotropic case A = 1 is plotted for comparison. In this case, the Young’s modulus and Poisson ratio considered at the scale of the β single crystal are 69.4 GPa and 0.35, respectively.

Since the texture is random, the elastic Young’s modulus is the same for A = 1, 2.4, and 3. However, the predicted transition from the elastic domain to the viscoplastic one is done earlier for A = 1. As a result, a longer transition between elastic and viscoplastic states is observed. Indeed, as expected by the self-consistent model, the stress–strain curves for A = 1 achieve the same viscoplastic asymptotic state as A = 2.4 and 3 when the elastic strains become negligible (i.e., at large strains ~10% and ~15% for strain rates equal to $2\times 10^{-4}\;s^{-1}$ and to $2\times 10^{-3}\;s^{-1}$, respectively).

**Figure 3:**
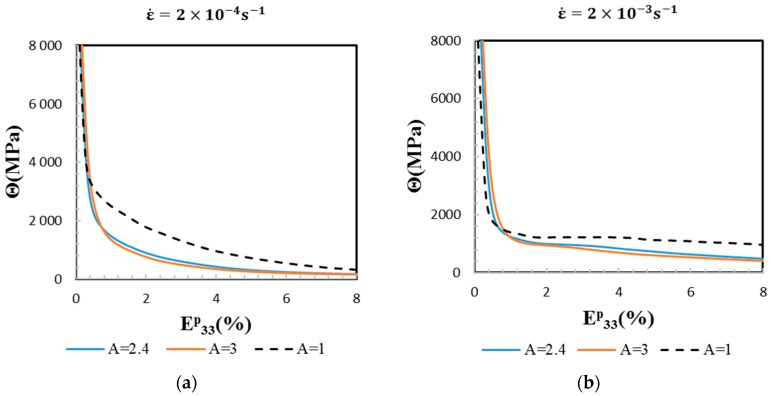
Comparison of overall strain-hardening rate evolutions as a function of macroscopic strain for different anisotropy factors (A = 1, 2.4 and 3) and at two tensile applied strain rates $\left(2\times 10^{-4}\;s^{-1}\;\left(a\right)\;\mathrm{and}\;2\times 10^{-3}\;s^{-1}\left(b\right)\right)$.

In addition to the macroscopic elasto-viscoplastic responses, the evolution of the overall tensile strain-hardening rate ($\Theta ={d\Sigma }_{33}/{\mathrm{dE}}_{33}^{p}$) is plotted as a function of the macroscopic tensile plastic strain. From Figure 3, it is clear that both elastic anisotropy and applied strain rate have an influence on the strain-hardening behavior. At $\dot{\varepsilon }=2\times 10^{-4}\;s^{-1}$, the predicted strain-hardening rate exhibits a smooth continuous decrease. At $\dot{\varepsilon }=2\times 10^{-3}\;s^{-1}$, the predicted evolution is less regular, even if there is no increase of the strain-hardening rate. The breaks into the steady decrease of Θ are due to the more important activation of slip systems in the course of plastic deformation, which is given by the threshold type law used for the viscoplastic flow rule (Equation (7)). The differences observed on overall strain-hardening rates between A = 2.4 or A = 3 and A = 1 are due to elastic incompatibilities and their coupling with plastic incompatibilities. Indeed, only plastic incompatibilities are present for A = 1. The coupling between elastic and plastic incompatibilities in the elastic-viscoplastic transition modifies the plastic flow stresses at the scale of slip systems. Hence, most slip systems become active sooner in the anisotropic case compared to the isotropic one, which explains the stronger decrease of Θ in the anisotropic cases A = 2.4 and 3.

### 3.3. Local Mechanical Fields Analysis

**Figure 4:**
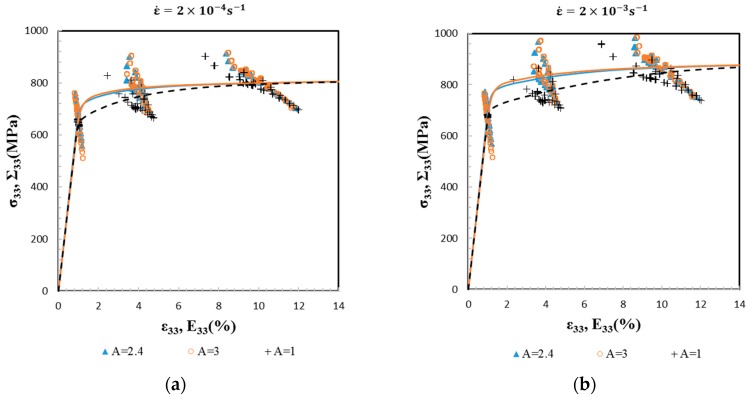
Local stress component $\left(\sigma_{33}\right)$ as a function of local strain component $\left(\varepsilon_{33}\right)$ at 1%, 4%, and 10% of macroscopic strain for three anisotropy factors (A = 1, 2.4 and 3) (cloud of dots) at two strain rates $2\times 10^{-4}\;s^{-1}\left(a\right)\;\mathrm{and}\;2\times 10^{-3}\;s^{-1}\;\left(b\right)$. The macroscopic stress–strain responses (Σ_33_ vs. E_33_) are plotted as references (solid lines).

**Figure 5:**
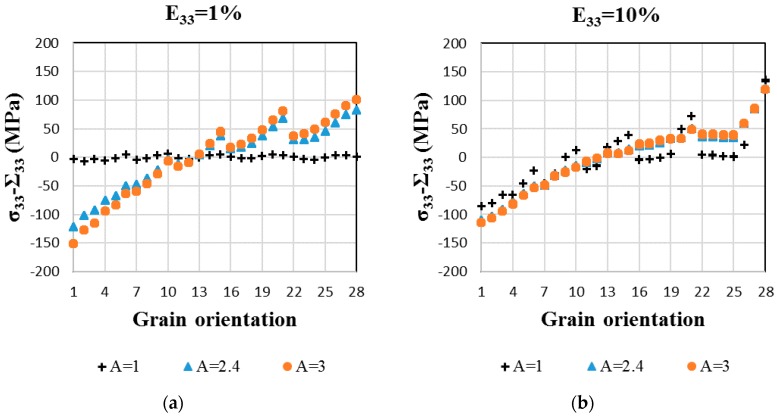

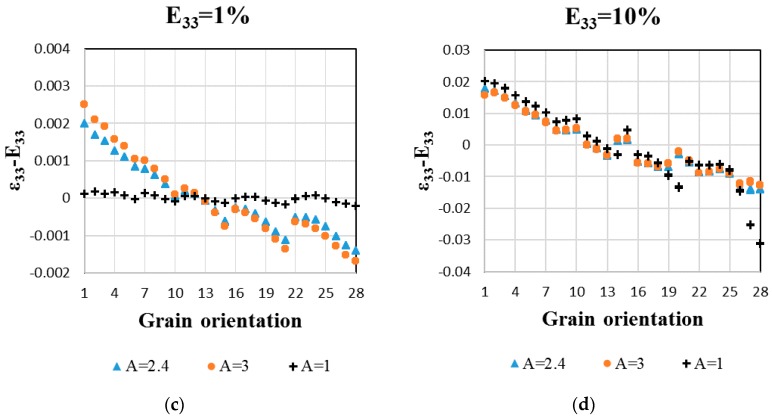
Incompatibility stresses (**a**,**b**) and incompatibility strains (**c**,**d**) (dimensionless) for grain orientations 1 to 28 at 1% and 10% macroscopic strains. The applied strain rate is $\dot{\varepsilon }=2\times 10^{-3}\;s^{-1}$. Inverse pole figure associated with the tensile axis, which defines the studied 28 grain orientations, were reported in Figure 1a.

Compared to the macroscopic results, the local behavior depends even more on the elastic anisotropy. Figure 4 shows at the two applied strain rates and for the three anisotropy factors A = 1, 2.4 and 3, the local stresses and the local strains of all of the grains at 1%, 4%, and 10% of macroscopic strain. For the isotropic case A = 1, the elasticity is homogeneous throughout the aggregate, and hence, differences between grains arise only at the onset of plastic deformation in the material. In Figure 4, it is seen that the cloud of dots related to a same simulation forms a line at a given macroscopic strain. The slope of these lines decreases with macroscopic strain, meaning that the range of local strains extends with macroscopic strain. This is in contrast to a simple Taylor’s model of uniform strain, which would lead to vertical lines at any strains. For the anisotropic cases, the range of local stresses is at its maximum toward the end of the elastic regime and in the elastic-viscoplastic transition. The cloud of dots obtained with A = 2.4 and 3 are relatively close to each other. The difference between A = 2.4 and 3 is observable when comparing local stress or local strain for a same specific grain orientation, such as <100>-oriented grains parallel to the loading direction (see Figure 5).

In Figure 5, incompatibility stresses, which are defined as the difference between the mean local stress (in the grain) and the macroscopic one $\sigma_{33}-\Sigma_{33}$, and incompatibility strains, which are defined as the difference between the local strain and the macroscopic applied one $\varepsilon_{33}-E_{33}$, are plotted as a function of the grain orientations. At 1% macroscopic strain, incompatibility stresses and incompatibility strains obtained for A = 3 are larger in magnitude than the values obtained for A = 2.4, whatever the grain orientation. Incompatibility stresses and incompatibility strains are maximal for grain orientation 1, which is close to a <100> direction, and are minimal for grain orientation 13, which is close to a <529> direction. However, the maximal local stress occurs for grain orientation 28, which is close to a <111> direction. Indeed, in this case, incompatibility stresses have a positive contribution, whereas the contribution is negative in <100> grains (i.e., the local stress is lower than the applied stress). These incompatibilities are due to elastic anisotropy and depend on the grain orientation.

With increasing deformation, plastic strain incompatibilities arise primarily because plastic strains become much larger than elastic strains. Therefore, the incompatibility stresses and incompatibility strains of different anisotropy factor A will all converge to the same set of values. In Figure 5b,d, it can be seen that this convergence is not completely achieved at 10% of macroscopic strain. The full convergence is actually obtained at a macroscopic strain of 15% (not shown).

### 3.4. Effect of Anisotropic Elasticity on Relative Slip Activities in 100% β-Ti

Thanks to the micromechanical model, the contribution of the different slip families to the overall plastic deformation can be deeply analyzed. The relative activity of a slip family can be studied from the definition of the α parameter, which is defined as:

(8) $$\alpha =\frac{\sum_{g=1}^{ng}\sum_{s=p}^{q}f_{g}\left|{\dot{\gamma }}_{g}^{s}\right|}{\sum_{g=1}^{ng}\sum_{s=1}^{ns}f_{g}\left|{\dot{\gamma }}_{g}^{s}\right|}$$

In Equation (8), $f_{g}$ is the grain volume fraction, $n_{g}$ is the total number of grains, p and q are the first and the last slip system number of the slip family, and $n_{s}$ is the total number of slip systems (see Table 2).

**Figure 6:**
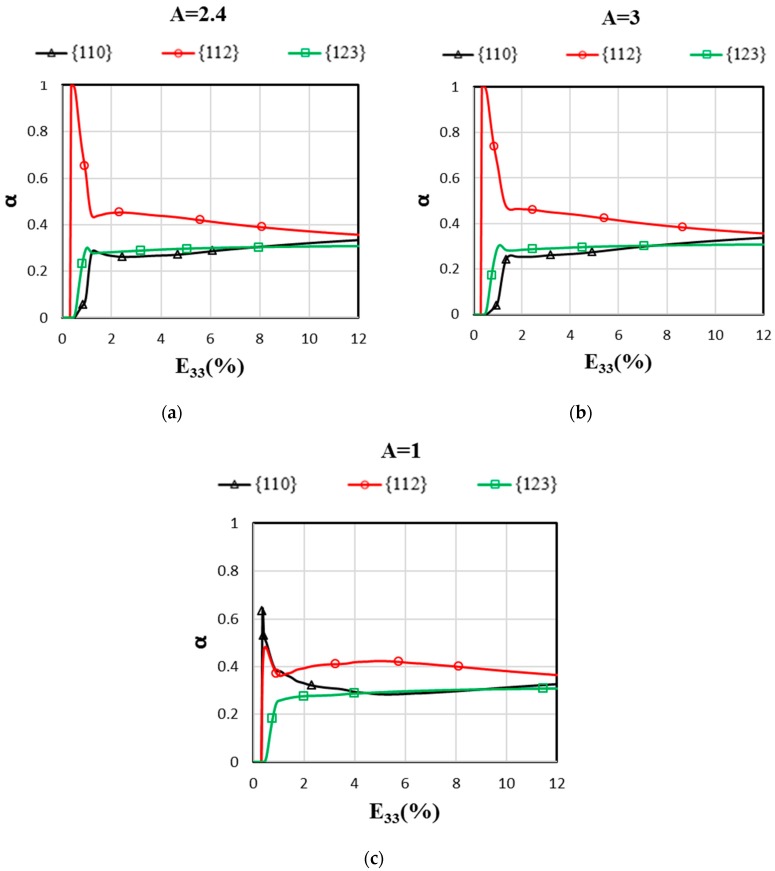
Relative slip plane family activities (dimensionless) as a function of the macroscopic strain predicted by the EVPSC model for A = 2.4 (**a**), 3 (**b**), and 1 (**c**). The applied tensile strain rate in the X_3_-direction is $\dot{\varepsilon }=2\times 10^{-4}\;s^{-1}$.

**Figure 7:**
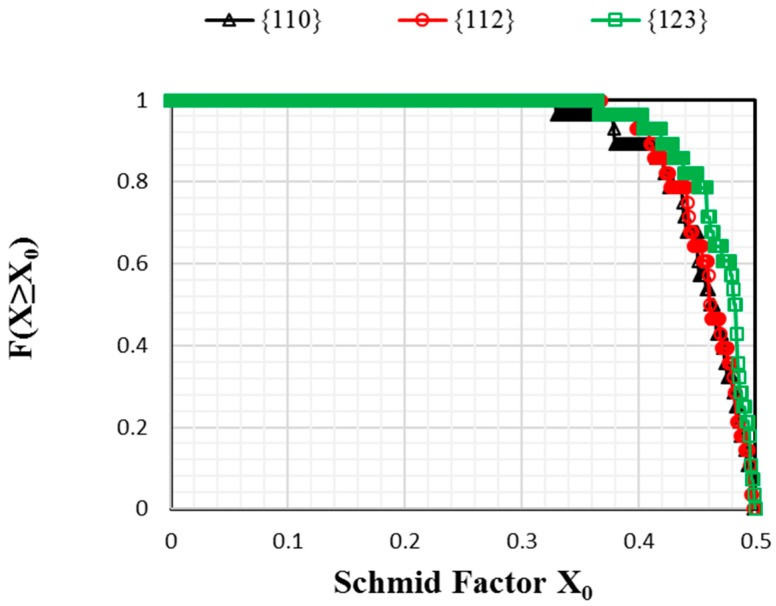
Cumulative distributions F($X\geq X_{0}$) of maximum Schmid factors in grains distributed with an isotropic texture. The representation of the distributions is chosen in such a way that the ordinate of a point $X_{0}$ gives the fraction of grains with the Schmid factor $X_{0}$ or higher.

The evolution of the parameter α related to slip families {110}, {112}, and {123} is plotted on Figure 6 for A = 1, 2.4, and 3. The value of α depends mostly on the texture, the viscoplastic parameters that are specific to slip families, and the intergranular stresses due to elastic/plastic incompatibilities predicted by the EVPSC model. For the present simulations, the initial texture is random, and thus, there is almost no difference in the cumulative distributions considering a classic Schmid factor analysis between the three slip families (Figure 7). From the Schmid factor analysis, it can be noticed that the initial texture should be slightly favorable for {123} systems, and that the distributions of families {110} and {112} are almost equal (Figure 7). However, it is reported from Figure 6 that the evolution of {112}<111> systems activity predicted by the EVPSC model is largely predominant at the onset of plastic deformation. Then, it decreases rapidly while {123} systems are activated first, and are followed closely by {110} systems. The contribution of the {112} systems remains predominant throughout the loading, although the relative contributions of the three families are very close at high strains.

Following some trends reported in the literature on body-centered cubic metals [34], the viscoplastic parameters are chosen to be identical for {110} and {112} families, and the {123} family has higher values of $r_{0}$ and c (see Table 2). Together with Figure 7, this explains why {123} slip systems are activated after the quasi-simultaneous activation of {110} and {112} systems in the isotropic elasticity case (A = 1) where intergranular stresses are negligible below 2% (see Figure 4). On the contrary, in the anisotropic cases (A = 2.4 and 3), the intergranular stresses that depend on the elastic anisotropy of the β-phase give clear advantage to the activation of the {112} slip family in comparison with the {110} slip family. While the literature shows that there is a strong competition between the activations of the {110} and {112} slip planes as a function of temperature in body-centered cubic metals [34], the present findings need to be checked by slip analysis in a future experimental study. In addition, it was proved from slip analysis and analytical/numerical calculations in face-centered cubic metals that elastic anisotropy and induced incompatibility stresses may drastically change slip activity in the microplastic regime that is observable in polycrystals and bicrystals [35,36].

## 4. Conclusions

The impact of the elastically anisotropic β-phase on the tensile behavior of a fully β-phase Ti-17 was studied with an affine extension of the self-consistent scheme for elasto-viscoplastic polycrystals (EVPSC) based on the translated fields (TF) method. Due to the uncertainties on the β-phase single-crystal elastic constants (SEC), two sets of SEC with different Zener anisotropy factors (A = 2.4 and 3) were considered in the simulations, as they both give effective Young’s moduli close to the measured one for a 100% β-Ti-17 polycrystal. The numerical results are in good agreement with the experimental data. The main conclusions of this work are summarized as follows:

1. At the macroscopic scale, the two sets of β-phase SEC well predict the tensile elasto-viscoplastic behavior of a randomly oriented and equiaxed β Ti-17 at two strain rates by the EVPSC model. This shows the relevancy of the model for the prediction of the material’s hardening and strain rate sensitivity;
2. In contrast, at the grain scale, the two sets of SEC lead to the scattered incompatibility of stresses and strains. These incompatibilities increase in magnitude with the Zener factor A and depend on the grain orientation. The grains oriented with their <100> direction parallel to the tensile axis are more sensitive to the anisotropy factor A (Figure 5). The local mechanical fields analysis demonstrates that considering only the macroscopic behavior is not sufficient to evaluate the effect of the elastic anisotropy of the β-phase;
3. Lastly, it is shown that the elastic anisotropy of the β-phase can affect the slip activities of a fully β-phase microstructure. Contrary to the isotropic elastic case, {112} <111> slip systems are clearly predominant at the onset of plasticity in the simulations with A = 2.4 and 3 (Figure 6).

## Figures and Tables

**Table 1:** β-phase single-crystal elastic constants (SEC) considered in this study, Zener elastic anisotropy factor (A) and effective Young’s modulus (EYM) computed with the purely elastic self-consistent scheme [32,33] for 100% β-microstructure assuming equiaxed grains and random texture.

Ti-Based Alloy	SEC (GPa)			A	EYM (GPa) of 100% β (Calculated)
	C_11_	C_12_	C_44_		
Ti-17 [4]	100	70	36	2.4	69.4
Pure [6]	134	110	36	3	66.6

**Table 2:** Viscoplastic parameters of the β-single crystal for each slip system family.

Slip Family	n	K(MPa·s^1/n^)	r_0_(MPa)	c(MPa)
{110} <111> 12 slip systems	20	300	113	200
{112} <111> 12 slip systems	20	300	113	200
{123} <111> 24 slip systems	20	300	123	400
